# Vibration or Transcutaneous Tibial Nerve Stimulation as a Treatment for Sexual Dysfunction in Women with Spinal Cord Injury: Study Protocol for a Randomized Clinical Trial

**DOI:** 10.3390/ijerph19031478

**Published:** 2022-01-28

**Authors:** María del-Carmen Díaz-Ruiz, Rita-Pilar Romero-Galisteo, Beatriz Arranz-Martín, Rocío Palomo-Carrión, Sara Ando-Lafuente, Cristina Lirio-Romero

**Affiliations:** 1 Department of Physiotherapy, Aranjuez Nursing Home Center, 28300 Madrid, Spain; mcarmen.diaz4@alu.uclm.es; 2 Department of Physiotherapy, Faculty of Science Health, University of Málaga, 29016 Málaga, Spain; 3 Department of Nursing, Physiotherapy and Occupational Therapy, Faculty of Physiotherapy and Nursing, University of Castilla-La Mancha, 45071 Toledo, Spain; beatriz.arranz@uclm.es (B.A.-M.); rocio.palomo@uclm.es (R.P.-C.); profesor.sando@uclm.es (S.A.-L.); cristina.lirio@uclm.es (C.L.-R.)

**Keywords:** spinal cord injuries, women, pelvic floor disorders, sexual and gender disorders

## Abstract

Background: Women with spinal cord injuries usually suffer from sexual dysfunction, such as alterations during arousal and an increase in the time to reach orgasm. However, little evidence has been found on its physiotherapeutic approach, as well as poor adherence to the latter. The aim of this study is to determine the effectiveness of two interventions to improve sexual dysfunction: the application of genital vibration and transcutaneous tibial nerve stimulation. Methods: This is a randomized clinical trial that will recruit 54 women who, one year after a spinal cord injury, suffer from sexual dysfunction associated with the latter. The participants will be randomized to three groups: (a) intervention group 1 treated with transcutaneous tibial nerve electrostimulation (*n* = 18), (b) intervention group 2 treated with genital vibration (*n* = 18), and (c) a control group (*n* = 18). The treatment time will be 12 weeks. Adherence to the treatment will be evaluated, as well as the effectiveness of the treatment, through the Female Sexual Function Index, the Sexual Quality of Life-Female questionnaire, quantitative sensory tests, and the improvement reported by the patient in terms of arousal and orgasm. The evaluations will be carried out before the treatment, at the end of the treatment and 3, 6 and 12 months after the end of the treatment.

## 1. Introduction

Neurological alterations after the Spinal cord injury (SCI) tend to produce changes in the sexual area. Four phases are differentiated in sexual function: arousal, plateau, orgasm and resolution [1]. The spinal cord suffers a nerve interruption that leads to different motor and sensory losses under the lesion level, which can generate a permanent disability [2]. About 80% of SCIs in Spain occur in the age range of 16–45 years [3]. Although SCI is less frequent in women (20%) [4], it is worth highlighting that, in the last years, the percentage of women with SCI has increased [5] and that 88% of these women experience sexual dysfunction (SD). This percentage, compared to the one found in the general female population (37%), is significantly higher [6], with its consequent impact at the personal and social level, and thus on the quality of life.

In addition to the motor and sensory deficits, the main autonomous alteration after SCI is autonomic dysreflexia, which occurs namely in injuries above T6 [7]. Autonomic dysreflexia can be related to some treatment and process of pelvic floor dysfunction.

Some women with SCI, once they are over the phase of spinal shock, can experience arousal through tactile stimuli directly applied on the genitals (reflective arousal), which remains unaltered if there is no affectation of the sacral spinal segments and peripheral sensitivity transmitters [8]. Both reflective and psychogenic arousal is observed in women with incomplete SCI [9]. At the peripheral level, sexual function can also be affected depending on the level and complexity of the SCI [10].

With respect to orgasm, the capacity to have it remains unaltered in women with intact sacral arch [11], although it has also been demonstrated that it can be induced through the vagus nerve [12]. In these cases, there is an increase in the time needed to reach an orgasm. Together with these physiological alterations, SD also tends to be associated with anxiety and depression in patients who present it, since sexuality is an essential part of their quality of life after the phase of spinal shock is over [13], and it is initially left aside in the rehabilitation process [8].

The literature shows improvements in SD in women through the application of genital vibration [14,15] or transcutaneous tibial nerve stimulation (TTNS) [16,17]. With respect to transcutaneous peripheral neuromodulation through the tibial nerve (L4—S3) [18], this presents an area of easy access to such nerve, which shares the same metameric area with the somatic and parasympathetic innervation of the pelvic floor (S2—S4) [19]. It is believed that the stimulation of its sensory afferent fibers blocks the abnormal afferences of this nervous region [20]. Therefore, through this neuromodulation, it is possible to regulate or modify the nerve activity on the different structures [21] and balance the excitatory and inhibitory impulses that produce improvements in vaginal arousal and lubrication [16].

Regarding vibrators, the literature shows two uses: (1) applied in bursts, after which there is an increase in blood flow associated with arousal; and (2) continuous vibration, which stimulates the high sympathetic explosion related to orgasm [22], also resulting in a better tissue perfusion, which can help to reduce muscle tone and increase relaxation [23].

It is important to propose treatment options for SD in women after SCI, since, currently, only educational programs are provided in most cases at late stages, and the patients refer an important deficit of information and treatment of SD [24]. Furthermore, due to the heterogeneity of levels and complexity of SCIs, this study will analyze the effect of two possible interventions plus a control group. None of these two propositions of intervention have presented any adverse effect [25,26].

We hypothesize that both types of stimulation, combined with educational programs, improve the evaluated sexual dysfunction symptoms measured through the Female Sexual Function Index (FSFI) and the Sexual Quality of Life-Female (SQOL-F) questionnaire. Particularly, the application of low-frequency genital vibration improves orgasm and TTNS improves arousal in women with SCI. The aim of the present study is to evaluate the effectiveness on FSFI and SQOL-F of an educational program plus TTNS and vibration in women with SD after SCI.

## 2. Materials and Methods

A protocol of a randomized clinical trial is presented with a blind randomization in the evaluation (C.L.-R.) and in the data analysis (B.A.-M.). The present protocol was registered in the platform https://register.clinicaltrials.gov/ (accessed on 16 November 2021) (NCT05122325). The study will be carried out according to guidelines and recommendations established for protocols by the SPIRIT statement [27].

The study is aimed at women with incomplete SCI, after which they had presented some type of SD. The candidates who participate in the study must meet the following inclusion criteria: women with American Spinal Injury Association or ASIA impairment B, C or D incomplete SCI (with preservation of the sacral arch and non-absent sensory evaluation of the lower dermatomes) below T6; aged 18–60 years without menopausal values of the follicle stimulating hormone (31–134 U/L) in a normal analysis; who present some type of SD secondary to SCI with at least 12 months of evolution; who live in Spain and are willing to go to the center of evaluations and treatments; and who sign the informed consent.

On the other hand, the study will exclude those women with active pregnancy and those who present pre-existing pathologies in the genital area, genital malformation, previous neurosurgery that affects the capacity of genital response, SD prior to the SCI, pressure ulcers, severe medical disease or any type of pathology in which the use of low frequency is contraindicated. In addition, the study will also exclude those with a psychiatric disorder or dependency on narcotics, and those who use selective serotonin reuptake inhibitors, antipsychotics or other drugs that affect the sexual response.

Regarding the sample size predicted for this study, 15 subjects were estimated for each group, with an effect size of 1.1, Df 28, α = 0.05, power (1 − β) = 0.80 (1 − β err prob) = 0.80u. Moreover, 20% more women will be recruited in order to consider the possible heterogeneity of the group and participants lost during the course of the study. Thus, a total of 54 women (18 women per group) will be recruited.

The project will be advertised in social networks, as well as in all the entities that belong to the ASPAYM (Association of people with spinal cord injury and other physical disabilities) in Spain.

**Figure 1:**
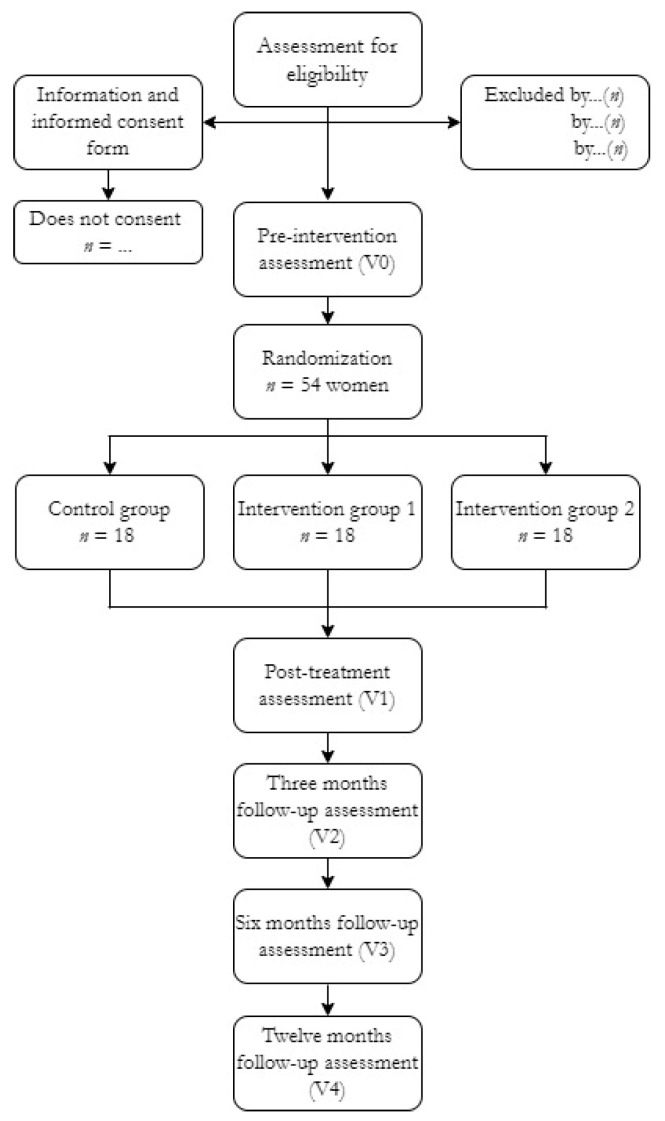
Flow chart of study participants.

The participants will be selected by non-probabilistic sampling from among those women who will reply to the advert and those associated with ASPAYM, who will be called for an interview in the University of Malaga or in the University of Castilla-La Mancha. The women who meet the inclusion criteria described above and sign the informed consent will be randomized by the Spanish version of the OxMar software [28] to either the control group (*n* = 18), the TTNS group (*n* = 18) or the vibration group (*n* = 18). Such distribution will be known to the lead researcher (M.d.-C.D.-R.), who will oversee and indicate to each participant, in a closed envelope, the group they will be assigned to (Figure 1).

### 2.1. Interventions

Both the evaluations and the treatment will be conducted in the care unit of the University of Malaga and University of Castilla-La Mancha (Spain). To facilitate the adherence of the participants, a weekly follow-up will be performed by two physiotherapists (M.d.-C.D.-R. and S.A.-L.) via phone call, through which the participants will be reminded of their appointments. The different interventions will be carried out by two physiotherapists (M.d.-C.D.-R. and S.A.-L.).

#### 2.1.1. Intervention Group 1: Transcutaneous Tibial Nerve Stimulation

**Figure 2:**
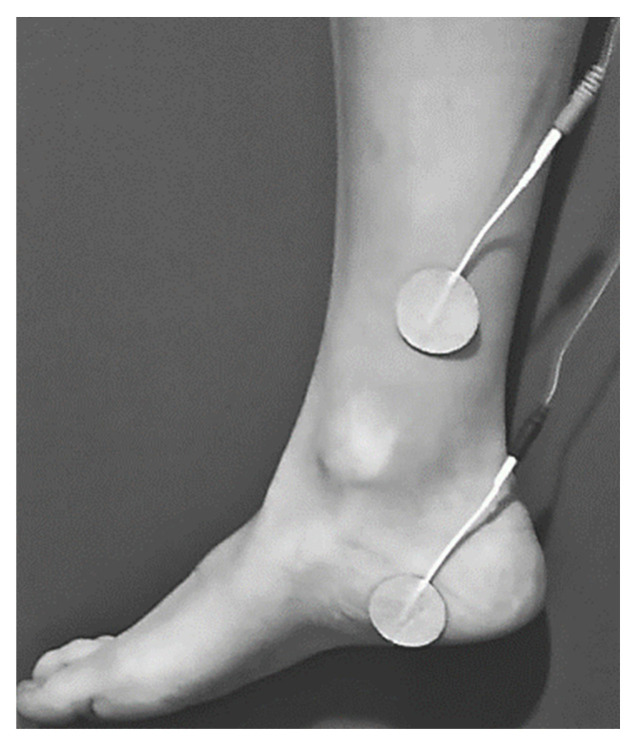
Transcutaneous tibial nerve stimulation (TTNS) electrode placements.

The women randomized to the TTNS intervention group will receive 12 stimulation sessions using a TENS^®^ EMS NMS60 device TENS, Madrid, Spain). These sessions will last 30 min each and will be conducted once per week at 10 Hz, with a pulse width of 200 μs and an intensity of 0–100 mA, which will be individually adjusted to each participant. Four electrodes will be placed: the first electrode 4–5 cm cephalad to the internal malleolus; the second electrode on the calcaneus area (Figure 2). Initially, the intensity will be gradually increased until the first toe shows flexion or abduction (motor threshold) or until the rest of the toes show flexion. Once the stimulation of the tibial nerve is ensured, the frequency will be raised to 10 Hz and the intensity will be lowered until the sensory threshold is reached [29].

#### 2.1.2. Intervention Group 2: Genital Vibration

The women randomized to the genital vibration intervention group will be trained in the use of the Ferticare 2.0^®^Medical Vibrator (Multicept, Rungsted, Denmark) accepted by the Food and Drug Administration), and each participant will be provided with one of these devices. Initially, a practical session will be carried out with them, explaining to them that the frequency of use will be 70 Hz, with an amplitude of 1.5 mm. It will be recommended to them to gradually increase its use in time, with a limit of 30 min per day. The intensity will be increased progressively until small vibrations are found [15]. The vibrating head can be placed in any of the 5 genital points innervated by the pudendal nerve (clitoris, bilateral labia majora and bilateral perineum), using the minimum pressure required to maintain the position [25]. They will be asked to use it once per week, before or during a sexual encounter, and they will also be required to record its use and any doubt related to it. Every week, a 30-min visit will be performed to check how they are using it and solve any doubts that may emerge.

#### 2.1.3. Control Group: Sham-Transcutaneous Tibial Nerve Stimulation

The guideline of application will be the same as that for the TTNS intervention group, but with the device switched off. The electrodes will be placed, and their location will be assessed to ensure that they are on the desired area. Once the intensity is lowered to reach the sensory threshold of the treatment, they will leave it at an intensity of 0. Then, the device in sham TTNS will be switch on for 30 s at the beginning, switch off for 20 min, and switch on for 30 s before stopping [30].

#### 2.1.4. Common Guidelines for the Three Groups

The interventions will be conducted throughout a period of 12 weeks. Prior to the interventions, the participants will be briefed on and given a handout with basic information and guidelines, common to all participants, focused on rediscovering sexuality after the SCI [31] (Table 1).

### 2.2. Outcome Measurement

The outcome will be the improvements obtained in FSFI, SQOL-F, sensory tests and specific improvement reported in orgasm and arousal.

The FSFI, whose use is supported by the Autonomous Standards Committee for the evaluation of sexual function in women with SCI [9], has been translated to and validated in Spanish [32], showing validity, internal consistency in all its domains (Cronbach’s Alpha of 0.745 in arousal and 0.753 in orgasm) and a test-retest reliability of ICC 0.96. This questionnaire consists of 19 questions, which are grouped into 6 domains. The score of each domain is multiplied by a factor and the final result is the arithmetic sum of the domains. The higher the score, the better the sexuality.

The changes in the quality of life will be objectified through the SQOL-F [33], which also showed good validity and reliability. The questionnaire was designed to measure the impact of SD on the quality of life [34]. This questionnaire consists of 18 questions with a response scale of 6 options. The sexual quality of life will be considered as follows: poor for a score of 18—51 points, moderate for 51–84 points, and good for over 84 points [35]. Additionally, the quantitative sensory tests will be used, whose validity has been proved in neurogenic female SD [36]. These tests consist in quantitatively measuring the sensory function in a non-invasive manner. Using a software-controlled probe, vibration is applied in a precise and controlled manner to the clitoral and vaginal areas, establishing the sensory thresholds of each participant, which are then compared with normal values established in previous studies to determine the presence of hyposensitivity or hypersensitivity and correcting the data for age.

Among these, previous studies have reported that vibratory stimuli are the most suitable to evaluate the sexual area [36]. Initially, the obtained results will be compared with the normal values obtained in previous studies [37], considering them as altered if they are outside of the confidence interval assigned to each age group, and, subsequently, with the previous values of each participant.

### 2.3. Data Collection

To preserve the confidentiality of the participants’ data, the researchers commit to complying with Organic Law 3/2018, of December 5th, on the protection of personal data and the Royal Decree that enforces it (RD 1720/2007). The participants will receive a code that will be related to the personal data only in a coding sheet. The evaluations will be conducted by a blinded researcher (C.L.-R.), who will initially collect the data of the participants in a data collection sheet: age, body mass index, data and type and level of spinal cord injury, pathologies or complications derived from the injury, allergies, pregnancies, type of deliveries and other diseases. Then, the participants trained by this researcher will complete the FSFI questionnaire, the SQOL-F, and the quantitative sensory test at the beginning of the treatment (V0, before the participants are randomized to any of the 3 groups), after the treatment (V1), and at 3 months (V2), 6 months (V3) and 12 months (V4) after the end of the treatment. C.L.-R. will be previously trained to standardize the measurements and will be blinded throughout the entire study to the randomization of the participants.

### 2.4. Data Analysis

Firstly, a test will be performed to determine the efficacy of the randomization of the baseline characteristics of the participants in the 3 groups through the Shapiro-Wilk test and normal distribution diagrams.

Secondly, we will establish the rate of adherence of the different participants randomized to each of the groups and their percentage of abandonment, comparing their effect through the *t*-test or Mann-Whitney *U*-test to determine whether the groups differ in the demographic variables in V0.

Thirdly, the comparisons to determine the effect of the interventions on FSFI, SQOL-F and the quantitative sensory tests in different time points will be analyzed with ANOVA, or Kruskal-Wallis for non-parametric samples, using the Bonferroni method for the multiple comparisons between different evaluation time points. The results will be considered statistically significant if *p* < 0.05. Moreover, the differences between each evaluation after the intervention and the arousal and orgasm before the intervention will be recorded in binary variables of “improvement of arousal/orgasm”, with values 0 (same or lower arousal) and 1 (greater arousal). Thus, improvement in all variables will be compared with the rate of participants who increase their sensation in the three groups through the risk ratio and the χ^2^ test. The data analysis will be carried out using the SPSS software v.25.0 (IBM, New York, United States).

### 2.5. Ethical Issues

This intervention proposal has been sent for approval to the University of Malaga Clinical Research Ethics Committee (153/2021). The ethical principles of the declaration of Helsinki will be followed throughout the entire study. The confidentiality of the study data will be a priority.

## 3. Discussion

After evaluating the effectiveness of an educational program plus either intervention (TTNS or vibration) in women with SD after SCI, it is expected to observe improvements in sexual function and the quality of life in both intervention groups. However, considering the neuropathophysiology of the injury, differences in arousal and orgasm will be also expected.

Regarding sexual function, the sympathetic routes mediate psychogenic arousal and require intact thoracic-lumbar segments (T11—L2). It is fundamental that women have their sacral segment intact (S2—S4/5) in order to reach orgasm. However, despite complete SCIs, orgasmic sensations persist and are accompanied by predominantly sympathetic signs, followed by parasympathetic signs (after orgasm) [15]. Therefore, the application of low-frequency genital vibration, due to its hyperemia effect, can improve relaxation and, thus, orgasm in women with SCI [22,23]. On its part, TTNS has shown excitatory effects through sensory afferences [20]. Consequently, the aim is to balance the excitatory and inhibitory impulses (sympathetic and parasympathetic mediation), which results in a possible improvement of vaginal arousal and lubrication [16].

Most of the studies found in the literature on treatments to improve the sexual function refer to transcutaneous tibial nerve stimulation [38,39,40]. However, due to the risks derived from needle puncture and its possible rejection, transcutaneous stimulation is proposed, since an access route has been demonstrated with similar effectiveness and no adverse effect associated that can generate discomfort in the participants [41,42]. Moreover, the literature shows a majority of studies on SD in SCI with only males [43]. Nevertheless, a qualitative study reported that sexuality is still an important and valued aspect of the female identity after SCI [44]. The same authors assert that sexual activity, although altered, is still pleasing and gratifying. Therefore, it is essential to study different interventions that help women to reestablish their sexual activity and improve their quality of life after SCI.

Limitations: to obtain the sample, a non-probabilistic sampling will be conducted, which will reduce the representativeness of the population. In addition, adherence to the treatment has been a problem in previous studies with women with SCI, thus the participants will be reminded of their appointments via phone call or confirmation messages, for all three groups. The literature shows a high rate of abandonment in women with SCI, which can reduce the possibility of obtaining statistical significance [15].

Future research lines in women with SD after suffering SCI would involve the design of qualitative studies that gather the opinions and perceptions of women with SCI towards the intervention received. Furthermore, it would be useful to plan group interventions that allow drawing an enriched conclusion about the opinion of the study population, as well as education and support for women with SD. Authors should discuss the results and how they can be interpreted from the perspective of previous studies and of the working hypotheses. The findings and their implications should be discussed in the broadest context possible. Future research directions may also be highlighted.

## 4. Conclusions

This protocol will analyze, in a poorly studied field, interventions for sexual dysfunction in women after SCI to promote the social participation of the women included in this research. The randomized design will allow determining the specific field for improvement of each intervention in SD. The educational handout designed as a common guideline will benefit all the participants, regardless of the group to which they are assigned.

## Figures and Tables

**Table 1:** Educational handout common guideline.

New Sources of Excitation after Spinal Cord Injury	
Improving your sexuality after spinal cord injury	If you wish to have sex alone or with your partner, first of all, it is important to try to find positions that help you to manage your body’s spasticity. You can try this in your wheelchair or in the shower. On the other hand, we want to show you a different way to enjoy sexuality by rediscovering yourself; therefore, here we list two activities to do alone and/or with your partner.
Discover your erogenous zones	Sexuality is something natural in human beings and therefore, after the spinal cord injury, the brain has the capacity to adapt to the new situation, favoring the appearance of new erogenous zones or strengthening those it had before. Rediscover yourself and look for new areas of pleasurable sensation that will serve to initiate sexual arousal, which requires you to explore your whole body, although you can focus on your ears, nipples, neck and hair. With this exploration you can elaborate a body map of sensitive areas, classifying those with which you have response of sexual arousal. ^1^
Rediscover your body	To rediscover your body, you can use the somatosensory massage. It will last approximately 30 min and will be performed very slowly. It is recommended as an initiation to sexual intercourse. You can do it yourself, with the limitation that there will be areas that you do not cover, or with the help of your partner. It can be done in underwear or completely naked, as you feel more comfortable. Talcum powder or oil can be used in a pleasant environment with lights or scents that allow you to concentrate on the sensations of touching different parts of your body. Once you start, it is important to avoid interruptions.
Steps to follow in somatosensory massage	Lie on your stomach and touch all areas of your body to improve your overall proprioception. It is important to mention in a soft and pleasant voice the part of the body over which we are sliding our fingers. Start with the foot and very slowly go over all the skin without exerting pressure, then continue along the leg, thigh, genial area, buttocks, opposite leg, back, shoulder, arm, forearm, hand, fingers, neck and head. Afterwards, lie on your back and again touch the lower limbs, genital area, abdomen, breasts, upper limbs, neck and head. In the genital area, when facing upwards, it is important to focus on touching all the areas that make up the genital area: starting with the labia majora, labia minora, entrance to the vagina and finally removing the clitoral hood to gently caress the clitoris.

^1^ Greater (+++) or lesser (+) response of sexual arousal.

## Data Availability

Data will be held securely by the research team and may be available upon reasonable request and with relevant approvals in place.
